# PEPAMARKER: a multicenter cohort study protocol on predictive biomarkers of affective *vs*. non-affective trajectories in first-episode psychosis

**DOI:** 10.3389/fpsyt.2025.1702187

**Published:** 2026-01-14

**Authors:** Raphaël Terrisse, Christophe Lemey, Deok-Hee Kim-Dufor, Louise Miglianico, Florian Stéphan

**Affiliations:** Service Hospitalo-Universitaire de Psychiatrie Générale et de Réhabilitation Psychosociale 29G01 et 29G02, Unité de Recherche 7479 Soins primaires, santé publique et registre du cancer de Bretagne Occidentale (ER 7479 SPURBO), Centre Hospitalier Universitaire (CHRU) de Brest, Hôpital de Bohars, Brest, France

**Keywords:** biomarker, early detection, first episode psychosis (FEP), mood disorder, psychosis

## Abstract

**Background:**

Psychosis is a severe and disabling mental disorder with peak incidence in late adolescence and early adulthood. Following a first-episode psychosis (FEP), clinical trajectories diverge into affective psychoses or non-affective psychoses. At illness onset, differentiation between these trajectories is frequently impossible, which results in delayed treatment adaptation and increased relapse risk. Predictive biomarkers, particularly linguistic and inflammatory markers, may help refine early diagnosis and personalize care.

**Objective:**

The primary objective of the PEPAMARKER study is to develop a predictive model based on prosodic markers to identify affective *vs*. non-affective trajectories at 2-year follow-up of patients with first-episode psychosis.

**Methods:**

PEPAMARKER is a prospective, multicenter, minimal-risk study conducted in five psychiatric centers in France. A total of 217 participants aged 15–30 years with FEP will be enrolled and followed for 24 months. At baseline, data will be collected through clinical interviews (audio-recorded and transcribed), standardized rating scales, and inflammatory markers. Prosodic markers will constitute the primary predictors. Secondary predictors include syntactic/semantic features, inflammatory biomarkers, and clinical rating scales. The primary endpoint is affective *vs*. non-affective evolution of psychosis at 2 years. Statistical analyses and logistic regression models will be conducted along with the assessment of internal validity.

**Expected impact:**

The study aims to provide accessible predictive tools using clinical interview recordings and basic blood tests to improve early differentiation of psychosis trajectories, which is crucial to a timely treatment adaptation and a reduction in relapse risk.

**Clinical Trial Registration:**

https://clinicaltrials.gov/, identifier NCT05384392.

## Introduction

Psychosis is a frequent and severe mental disorder (1). Epidemiological data from the Social Epidemiology of Psychosis in East Anglia (SEPEA) study in England reported an incidence of 34 new cases per 100,000 person-years, and a peak between ages 16 and 19 was reported (2). Following a first-episode psychosis (FEP), two broad clinical trajectories can be observed: affective psychoses (approximately 17%) and non-affective psychoses (approximately 83%). A major clinical challenge is that at the onset of psychosis, it is often impossible to determine which trajectory a patient will follow. This diagnostic uncertainty delays adequate therapeutic decisions and increases the risk of relapses.

In recent years, advances in the field of computational psychiatry have had underscored the promise of linguistic biomarkers as potential predictors of illness trajectory (3–6).

Language production and comprehension allow interpersonal communication based on different linguistic components such as syntax, semantics, and phonology. When a speaker wants to express their thoughts, ideas, and/or feelings (semantics), they structure the necessary words (syntax) and utter the latter (phonology). A listener hears the sound and understands the utterance by processing the elements in the opposite direction (7). The study of language has the potential to provide a quantitative and objective measure of disorders. This approach has the advantage of acquiring a supplementary instrument in a non-invasive way to enhance the diagnostic and prognostic capabilities for patients afflicted with psychiatric disorders. The employment of machine learning facilitates a more sophisticated graphical analysis of language than that achievable with clinical scales (8, 9). Language deficits have been identified in a number of psychiatric disorders, most notably in patients suffering from mood disorders or schizophrenia (10).

As demonstrated by several studies, patients suffering from schizophrenia can experience difficulties finding the appropriate words to express themselves (11) and present lexical disorders (12). Their discourse is also often marked by disfluency, an abusive use of relative propositions (13), or abnormal prosodic features (14).

Specific speech characteristics of bipolar disorder remain a matter of debate (15). Some studies find general impoverishment of speech, with a lack of detail and references to oneself, especially during depressive phases (16).

Differences in language disorders affecting patients with mood disorders accompanied by psychotic features compared to those with non-affective psychoses can be shown. It is thus possible to identify linguistic markers for these pathologies (17). Some studies have found that alterations in syntactic structures and prosody could be more prevalent in psychosis than mood disorders with psychotic features (5, 18, 19). A study by Perlini et al. also suggested that syntax and verbal abilities were impaired in both affective and non-affective psychoses, but more frequently and severely in schizophrenic disorders (20). Illogicality would be a key difference between schizophrenic disorder and bipolar disorder (17). A systematic review emphasized the value of natural language processing (NLP) and machine learning in mental health prediction (8).

In parallel, biological markers have emerged, notably inflammatory markers. An immune dysfunction manifested by an increase in pro-inflammatory biomarkers and a decrease in anti-inflammatory biomarkers may be involved in the pathogenesis of psychiatric disorders in some individuals (21). Numerous studies have shown that depression, bipolar disorder, and schizophrenia are associated with immune response dysregulation. Metabolic syndrome is highly prevalent in schizophrenia, bipolar disorder, and major depressive disorder and may exacerbate or modulate inflammatory dysregulation. A large meta-analysis reported markedly elevated rates of metabolic syndrome across psychotic and mood disorders (22). In bipolar disorder, metabolic syndrome and its component factors have been shown to significantly influence the longitudinal course of illness, including relapse risk, chronicity, and functional outcomes (23). More recently, metabolic alterations have been linked to the activation of the kynurenine pathway and downstream inflammatory cascades in schizophrenia (24), further supporting the hypothesis that immune dysfunction in psychosis is embedded within a wider metabolic imbalance. It is also broadly documented that chronic inflammatory diseases are associated with a high rate of psychiatric comorbidity (25–27), frequently with an impact on mood (28). Finally, it is noteworthy that several antipsychotics and mood stabilizers have intrinsic anti-inflammatory properties (29).

The serum levels of inflammatory cytokines are elevated in individuals suffering from a major depressive disorder: increased levels of alpha cytokines TNF-α (tumor necrosis factor α), interleukin-6 (IL-6), and interleukin-1 beta (IL-1 β) (30, 31). A meta-analysis from 2011 (29) showed that cytokine levels did not decrease, even when patients experienced an improvement in their mood symptoms.

Some pro-inflammatory cytokines may also increase in patients with bipolar disorder. These include C-reactive protein (CRP), IL-1β, soluble interleukin-2 receptors (sIL2R), interleukin-4 (IL-4), IL-6, TNF-α, and TNF-α type 1 receptor (32). It has also been reported that the serum levels of these cytokines are mood dependent. Thus, high serum concentrations of IL-4, IL-6, TNF-α, sTNFR1 (soluble tumor necrosis factor receptor-1), CXCL10 (CXC motif chemokine ligand 10), and CXCL11 (CXC motif chemokine ligand 11) are observed in manic phases. Similarly, high serum concentrations of IL-6, IL-1β, CRP, TNF-α, sTNFR1 and CXCL10 are also found in depressive phases (33, 34). IL-6 and TNF-α levels appear to be directly correlated with disease severity (35).

Meta-analyses have shown that patients experiencing a first-episode psychosis and chronic psychosis have elevated blood levels of cytokines (36), including soluble interleukin-2 receptor (sIL2R), IL6, interleukin-8 (IL8), interleukin-10 (IL10), interferon-γ (IFNγ), transforming growth factor-β (TGFβ), TNFα, CRP, and hyperleukocytosis (37, 38). Recent findings support the hypothesis of distinct immuno-inflammatory profiles across FEP trajectories (39).

Within this context, the PEPAMARKER study seeks to combine clinical, linguistic, and biological data to build a predictive model of psychosis trajectory after a first episode. Early differentiation of affective trajectories would enable better targeted pharmacological and psychosocial interventions, which most likely reduces relapse rates and improves long-term functional outcomes (40).

## Methods and analysis

### Study design

The PEPAMARKER is a multicenter, prospective cohort study conducted in five psychiatric centers in France. The study is categorized as minimal risk and little constraint, in compliance with the French and international Good Clinical Practice guidelines. Based on Riley et al. (41), 217 participants are required to achieve sufficient statistical power to develop a multivariable prediction model with three candidate predictors, assuming 17% affective psychoses (2) and expected *R*² of 0.3 (42, 43). Participants with first-episode psychosis (FEP) will be included and followed for 24 months, with assessments at baseline, 12 months, and 24 months.

### Participants

The inclusion criteria are as follows:

✔ Ages 15 to 30 years
✔ Diagnosis of first-episode psychosis according to the DSM-5 criteria
✔ Ability to provide informed consent (for minors, consent obtained from at least one legal guardian)

The exclusion criteria are as follows:

✔ Introduction or recent adjustment (within 1 month) of antipsychotic, antidepressant, or mood stabilizer treatment
✔ Native language other than French
✔ Psychosis due to an organic disorder
✔ Substance-induced psychosis with severe dependence
✔ Intellectual disability
✔ Chronic inflammatory disease or immunomodulatory treatment: systemic inflammatory disorders (e.g., rheumatoid arthritis, lupus, inflammatory bowel disease, psoriasis), chronic inflammatory metabolic conditions (e.g., chronic type 2 diabetes with poor glycemic control, metabolic syndrome), and chronic inflammatory conditions linked to malignancy or chronic infections
✔ Pregnancy or breastfeeding since these states have been shown to induce profound immunological and hormonal changes that strongly alter inflammatory cytokine levels (44)
✔ Patients under guardianship, curatorship, or deprived of liberty

### Recruitment and consent

Eligible patients will be identified during hospitalization or outpatient visits. The investigators will explain the study objectives, procedures, and rights. Written informed consent will be obtained from all participants or guardians. Procedures and interventions.

### Study flow

Baseline assessment (T0):

- Socio-demographic and clinical data collection
- Audio-recorded clinical interview
- Clinical rating scales: PANSS (Positive and Negative Syndrome Scale), BPRS (Brief Psychiatric Rating Scale), CDSS (Calgary Depression Scale for Schizophrenia), MADRS (Montgomery–Åsberg Depression Rating Scale), Altman, YMRS (Young Mania Rating Scale), GAF (Global Assessment of Functioning), SF-36 (36-Item Short Form Survey), CGI-S (Clinical Global Impression Scale)
- Blood samples collected during routine care: inflammatory biomarkers (IL-1, sIL-2R, IL-4, IL-6, IL-8, TNF), C-reactive protein, vitamin D
- Biobanking: plasma (2 EDTA tubes, 6 mL each) and serum samples (2 SST tubes, 5 mL each) stored at the Biological Resource Center (CHU Brest). Blood samples are processed following the standard operating procedures of the certified biobank of the CHU de Brest, in accordance with the national NF S96–900 and ISO 20387 guidelines. In routine practice, EDTA tubes (for plasma) and SST tubes (for serum) are centrifuged shortly after collection, aliquoted under sterile conditions, and stored at –80 °C until batch analysis.

Follow-up assessments:

- 12 months (T1): Telephone or in-person diagnostic interview and treatment update
- 24 months (T2): Diagnostic interview, treatment update, and clinical rating scales

### Clinical rating scales

#### PANSS (Positive and Negative Syndrome Scale)

The Positive and Negative Symptom Scale is a 30-item scale, rated from 1 (absent) to 7 (extreme), which assesses psychopathological symptoms observed in patients with psychotic conditions, particularly schizophrenia. It allows scores to be calculated for three dimensions: positive symptoms (seven items), negative symptoms (seven items), and general psychopathology (16 items). Positive symptoms refer to an excess or distortion of normal functions (e.g., hallucinations), and negative symptoms represent a decrease or loss of normal functions. Its use is particularly indicated for determining a psychopathological profile, researching prognostic factors for progression, and evaluating the effectiveness of various therapeutic strategies (45).

#### BPRS (Brief Psychiatric Rating Scale)

The Brief Psychiatric Rating Scale is a rapid and highly effective assessment procedure to evaluate symptom changes in psychiatric patients. It includes a precise and comprehensive description of major characteristic symptoms. The factor analyses of the 18 items of the BPRS usually provide four or five underlying factors. The Diagnostic and Psychopathology Unit at the Clinical Research Centre in Los Angeles has developed an expanded version of the BPRS with 24 questions (46).

#### CDSS (Calgary Depression Scale for Schizophrenia)

The Calgary Depression Scale for Schizophrenia is designed to assess depression in this patient population. It consists of nine questions rated from 1 (absent) to 3 (severe), for which the effects of negative symptoms, psychotic symptoms, or treatment should be reduced, which is not the case with other scales (47).

#### MADRS (Montgomery–Åsberg Depression Rating Scale)

The Montgomery–Åsberg Depression Rating Scale is a scale used to assess the severity of depression in patients with mood disorders. It is also frequently used to measure changes brought about by depression treatment. It assesses the severity of symptoms in a wide range of areas such as mood, sleep, and appetite, physical and mental fatigue, and suicidal thoughts (48).

The scale consists of 10 items rated from 0 to 6:

- 0 to 6 points: The patient is considered healthy.
- 7 to 19 points: The patient is considered to have mild depression.
- 20 to 34 points: The patient is considered to have moderate depression.
- >34 points: The patient is considered to have severe depression.

#### Altman

The Altman Self-Assessment Scale is a short five-point self-assessment questionnaire (scored from 0 to 4) that can be useful for assessing the presence and severity of manic or hypomanic symptoms. As this scale is compatible with diagnostic criteria, it can be used effectively as a screening and diagnostic tool despite its brevity. Each of the five items is scored from 0 to 4 (49, 50).

#### YMRS (Young Mania Rating Scale)

The Young Mania Rating Scale comprises 11 items used to assess the severity of mania. The manic symptoms evaluated are euphoria, increased activity/motor energy, sexual interest, sleep, irritability, speech (rhythm and quantity), language disorders, thought content, behavioral alteration/aggressiveness, appearance, and lucidity.

The items 1, 2, 3, 4, 7, 10, and 11 are rated from 0 (no symptoms) to 4 (extreme symptoms). The items 5, 6, 8, and 9 are rated from 0 (no symptoms) to 8 (extreme symptoms). A precise description is given for each point on the scale. An overall score is calculated by adding up the scores for each item: [0–20] non-manic patient, [20–26] mild intensity, [26–38] moderate intensity, and [38 and above] severe intensity (51).

#### GAF (Global Assessment of Functioning)

The Global Assessment of Functioning scale is used to assess the severity of a mental illness. It determines the extent to which a person’s symptoms affect their daily life on a scale of 0 to 100. Its results help caregivers determine the level of care a person may need as well as the effectiveness of certain treatments (52).

#### SF-36 (36-item short form survey)

The SF-36 questionnaire is a standardized test to measure the quality of life. It contains 11 items rated from 0 to 6 (53).

#### CGI-S (Clinical Global Impression Scale) and CGI-I (CGI-Improvement)

The CGI-S and GCI-I scales are widely used in psychopharmacology. They enable clinicians to assess improvements in a patient’s condition over time after prescribing treatment or discontinuing it (54).

### Outcomes

#### Primary outcome

• Affective *vs*. non-affective evolution of psychosis at 2 years based on prosodic markers at baseline:

- Fundamental frequency (F0) variability
- Speech latency (mean response time and coefficient of variation)

#### Secondary outcomes

• Affective *vs*. non-affective evolution of psychosis at 2 years based on:

- Syntactic and semantic markers (8)
- Inflammatory biomarkers (39)
- Clinical scales

### Data collection and management

Data will be collected in electronic case report forms (eCRF). Interviews will be transcribed verbatim and analyzed using natural language processing (NLP) methods developed with IMT Atlantique (55). Biological samples will be processed and stored according to standardized protocols. Data confidentiality will be ensured through anonymization and compliance with the GDPR (General Data Protection Regulation).

### Statistical analysis

Statistical analyses will be performed with SAS 9.4 and R (version 4.0.4). All patients included will be analyzed according to the intention-to-treat principle.

- Primary analysis: Logistic regression models will evaluate the predictive value of prosodic markers (F0 variability, response latency). Model performance will be assessed using calibration and discrimination indices.
- Secondary analyses: Univariate logistic regressions will be conducted for syntactic/semantic markers, inflammatory biomarkers, and clinical scales. Variables with *p <*0.15 will be included in multivariate models. Stepwise backward elimination will retain predictors with *p <*0.05.

### Risk assessment

A methodological risk analysis was conducted to identify potential sources of bias in this multicenter prospective study. Selection bias may occur due to the inclusion and exclusion criteria, which could limit the representativeness of the broader first-episode psychosis population; this risk is mitigated through consecutive recruitment across centers using homogeneous eligibility procedures. Measurement bias is possible in both clinical assessments and speech-related measures, given differences in interviewing conditions, rather interpretation, or audio quality. To reduce this variability, all assessments rely on standardized scales administered by trained clinicians, and recorded interviews follow a uniform procedure, with biological samples processed according to established protocols in the certified biobank. Attrition bias is another anticipated challenge given the 24-month follow-up; the protocol allows a 12-month telephone visit and collects any available clinical information to limit missing diagnostic outcomes. Differences between centers may also introduce heterogeneity in patient profiles or clinical practices; this is addressed through harmonized procedures, central transcription of interviews, and regular monitoring ensuring adherence to the protocol. Finally, confounding factors such as psychotropic treatments, baseline symptom severity, or socio-demographic characteristics are documented systematically and will be accounted for in multivariable statistical modeling. The combination of these measures is intended to mitigate the primary methodological risks while maintaining the ecological validity of the study conducted in routine clinical settings.

## Discussion

The PEPAMARKER study addresses one of the central challenges in early psychosis care: the difficulty of distinguishing affective trajectories. Early differentiation is crucial, as treatment strategies and prognoses differ substantially between these subgroups.

A major strength of this study is its multimodal approach, combining clinical interviews, linguistic analyses, and inflammatory biomarkers. While linguistic markers have already shown promise in distinguishing between schizophrenia and bipolar disorder (5, 6), their predictive value in real-world FEP cohorts remains to be established. Similarly, inflammatory markers have been reported to differentiate affective psychoses (39).

Another strength lies in the ecological design: assessments are based on routine clinical interviews and simple blood tests, ensuring feasibility and acceptability in everyday psychiatric practice. The sample size (217 patients across the five centers) makes it possible to develop a robust multivariable predictive model (41).

Several limitations must, however, be acknowledged. First, the study only includes French-speaking participants. As linguistic markers are language-dependent, the predictive characteristics identified in French may not be generalizable to other languages. Second, the naturalistic type/kind/character of the cohort implies heterogeneity of treatments during follow-up, which may induce variability. The study also carries a risk of follow-up losses, as visits are spaced out over 2 years in a population often at the beginning of their care pathway where building a therapeutic alliance is a major challenge. Furthermore, the question of how inflammatory markers evolve over time may arise, as these are recently identified indicators that are still being explored scientifically. Another limitation relates to the symptomatology of first-episode psychosis and the fact that the patients included are not yet receiving treatment—that is, some patients may call into question their ability to consent or to be present for the initial assessments. This doubt or uncertainty likely exposes them to a selection bias, as the most severe forms are often treated immediately. From an ethical standpoint, it is worth mentioning the risk of de-subjectification of the patient, with the fear of a loss of clinical meaning in favor of computational approaches (56). Finally, other potential predictors such as brain imaging (57) are not included.

Despite these limitations, the present study is relevant to evaluate a wide range of clinical and paraclinical markers in routine care settings. These elements are easy to collect and minimally intrusive for patients. The proposed management is in line with usual practice, including the recording of a clinical interview. The only examination specific to the study is the search for inflammatory markers, which can, however, be easily integrated into the standard biological assessment carried out during first-episode psychosis.

The PEPAMARKER is expected to advance our understanding of early predictors of psychosis trajectory and generate accessible clinical tools for early differentiation. This project stands out due to the joint integration of three types of markers that are rarely combined: linguistic, clinical, and inflammatory. Automated language analysis, applied to first-episode psychosis, is an emerging and promising field (58). Few studies have sought to distinguish early affective and non-affective psychoses using biomarkers from a simple recorded clinical interview. The proposed multimodal approach provides an innovative way to explore the pathophysiology of first-episode psychosis and paves the way for objective standardized predictive tools that can be used soon after the patient is admitted.

The tools evaluated in this study, a recorded clinical interview and standard biological assessment, can be easily integrated into routine practice and do not require specialized technological resources other than a data processing pipeline currently undergoing automation. Their accessibility facilitates their deployment in various clinical contexts, including in facilities with limited resources.

In organizational terms, the potential benefits for the healthcare system are significant. Early differentiation between affective and non-affective psychoses is a major challenge, as it determines the speed and relevance of therapeutic strategies (40, 59). Reducing diagnostic uncertainty would limit relapses, repeated hospitalizations, and interruptions in treatment, with a direct impact on the workload of psychiatric services (60, 61).

## Ethics and dissemination

The study protocol was approved by the French Comité de Protection des Personnes (CPP Ile-de-France III, approval date: April 24, 2022). The trial is conducted in accordance with the principles of the Declaration of Helsinki, Good Clinical Practice (ICH-E6), and French regulations on research involving human participants. The sponsor is the University Hospital of Brest.

All participants will receive complete written and oral information about the study objectives, procedures, potential risks, and their rights to refuse or withdraw at any time without affecting their care. Written informed consent is required prior to enrollment. For minors who reach legal adulthood during the study, renewed consent will be obtained. No financial compensation is planned for participation.

Data protection complies with the European General Data Protection Regulation (GDPR, EU 2016/679) and the French Méthodologie de Référence MR-001 (CNIL approval, 2016). All study data are anonymized before analysis. The biological samples are stored in the certified Biological Resource Center.

The sponsor has subscribed to an insurance policy covering all potential risks related to study participation. The study is registered in a public clinical trial registry (ClinicalTrials.gov ID NCT05384392).

### Dissemination plan

Results will be disseminated through peer-reviewed publications and presentations at national and international conferences. Authorship will follow international guidelines (ICMJE). Negative and positive results will be reported. The goal of dissemination is to provide clinicians with validated predictive tools based on routine interviews and simple laboratory tests to improve the early differentiation of affective and non-affective psychoses.
